# Influence of *Plasmodium* gametocyte carriage on the prevalence of fever, splenomegaly and cardiovascular parameters in children less than 15 years in the Mount Cameroon area: cross sectional study

**DOI:** 10.1186/s12879-015-1290-4

**Published:** 2015-11-27

**Authors:** Irene Ule Ngole Sumbele, Orelien S. Mtopi Bopda, Helen Kuokuo Kimbi, Teh Rene Ning, Theresa Nkuo-Akenji

**Affiliations:** Department of Zoology and Animal Physiology, University of Buea, Buea, Cameroon; Department of Microbiology and Parasitology, University of Buea, Buea, Cameroon

**Keywords:** Malaria parasitaemia, Gametocytes, Cardiovascular parameters, Fever, Splenomegaly, Anaemia, Haematological values, Children

## Abstract

**Background:**

Cardiovascular parameters can be impaired by repeated infections with *P. falciparum*. This study aimed at investigating the influence of gametocyte carriage on; the prevalence of fever and splenomegaly, blood pressure, heart rate and haematological indices in children <15 years, in the Mount Cameroon area.

**Methods:**

A cross-sectional study was carried out, from February to July 2013. A child with axillary body temperature ≥37.5 °C was considered febrile and splenomegaly was investigated by palpation. Systolic and diastolic blood pressures as well as heart rate were assessed by non-invasive methods. Malaria parasites were detected and density assessed from Giemsa-stained thin and thick blood films. An auto haematology analyser was used to obtain complete blood count values such as haemoglobin (Hb), haematocrit (Hct), red blood cell (RBC) and white blood cell (WBC) counts, mean corpuscular volume (MCV), mean corpuscular haemoglobin concentration (MCHC), and mean corpuscular haemoglobin (MCH). Univariate analyses were used to examine influence of gametocyte carriage on fever and splenomegaly while, multiple linear regression models were used to evaluate influence of independent variables on the dependent variables.

**Results:**

Of a total of 454 children examined, malaria parasitaemia, fever, splenomegaly and gametocyte carriage were detected in 36.6, 21.6, 14.3 and 7.3 % of them respectively. Children who were asexual parasite and gametocyte positive (ASP + Gam Pos) had significantly highest (*P* = 0.03, *P* = 0.002) prevalence of fever and splenomegaly (39.4 %, 33.3 %) respectively than their aparasitaemic (AP) and asexual parasite positive (ASP Pos) equivalents (19.0 %, 10.9 % and 22.8 %, 16.9 % respectively). The presence of asexual malaria parasitaemia significantly influenced the MCV (*P* = 0.03), MCH (*P* = 0.03) and heart beats /min (0.03) while gametocytaemia significantly influenced the Hb (*P* < 0.001), Hct (*P* < 0.001), RBC (*P* < 0.001) and systolic blood pressure (*P* < 0.05).

**Conclusion:**

Gametocyte carriage significantly influenced the prevalence of fever, splenomegaly and some cardiovascular indices. In effect, children concurrently having asexual parasitaemia and gametocytes had significantly lower, Hct, Hb levels, RBC and platelet counts and systolic blood pressure.

## Background

Malaria is a febrile illness caused by protozoans of the genus *Plasmodium*. Globally, an estimated 3.3 billion people in 97 countries and territories are at risk of being infected with malaria parasite and developing disease [1]. Majority of patients present with fever, but fever alone remains a poor discriminator of malaria infection hence the need to confirm or refute the role of malaria in febrile presentation [2].

Malaria transmission from an infected human host to a female anopheline mosquito is mediated through highly specialized sexual-stage parasites, i.e., gametocytes. The gametocytes of *P. falciparum* hold a prominent place in the history of malaria [3]. Several factors including age, host immune response (including co-infection with other pathogens), host anaemia, insecticide spraying, and mass drug administration are likely to influence the appearance of gametocytes at presentation [3]. However, clinically immune infections often have lower gametocytemia as a result of having anti-parasite immunity, conferring protection against high-density parasitaemia [4].

Splenomegaly has been reported as one of the common pathologies among children with multiple parasitic aetiologies [5]. Splenomegaly which occurs in all forms of malaria with repeated attacks is the main clinical marker of endemicity in *P. falciparum* transmission areas [6]. A palpable spleen has been reported as an independent risk factor for anaemia [7, 8]. Individuals with either gametocytes or splenomegaly have a reduced mean haemoglobin (Hb) concentration when compared to those without [3, 9]. Though anaemia is generally used as a measure of the impact of malaria in patients [10], repeated infections with malaria might not only impair the level of Hb, but also other parameters of the blood and even the whole of the cardiovascular system [11]. Impairment of cardiovascular parameters as shown by tachycardia and hypotension [12–14] represents a major step towards malaria caused death. There is a paucity of knowledge on the malaria related alteration of cardiovascular indices in children in an endemic setting such as the Mount Cameroon area.

As an intraerythrocytic parasite of the fluid component of the cardiovascular system, *Plasmodium* induces haematological alterations involving major cell lines such as RBCs, leucocytes and thrombocytes which may lead to complications in malaria pathology characterised by splenomegaly, anaemia, thrombocytopenia and disseminated intravascular coagulation [13, 15]. This has been supported by Sumbele *et al*. [8, 16] who reported anaemia among children with malaria as a common haematological state in the Mount Cameroon area. Several studies [16–18] have been conducted on malaria in the Mount Cameroon area, but none on the impact on cardiovascular parameters. Furthermore, most of the cardiovascular findings reported so far are on severe/complicated malaria [11, 19–21]. The aim of this study was to investigate the additive effect of gametocyte carriage in children with malaria parasitaemia on the prevalence of fever and splenomegaly, on blood pressure, heart rate and haematological indices in children living in the Mount Cameroon area.

## Methods

### Study area and participants

The study was carried out in Muea, a semi-rural setting in the Mount Cameroon area. The coordinates of the study area ranged from altitude 540 m, latitude 04°10.464’N, longitude 009° 18.168’E to 556 m, 04°10.015’N and 009°18.009’E. The study area has been described in detail by Sumbele *et al.* [16]. The children who participated in the study were less than 15 years with or without symptoms of malaria. Children weighing <5 kg and those with severe malaria (unable to drink or breastfeed, vomiting more than twice in the preceding 24 h before presentation, recent history of convulsions, unconscious state or unable to sit or stand and other diseases requiring hospital admission) were excluded from the study.

### Study design

A cross-sectional study was carried out from February to July 2013, which included the rainy season (March - October) reported as the peak malaria transmission period in the Mount Cameroon area [22]. After obtaining ethical clearance, administrative and local authorizations for the study, the team with the help of the health personnel proceeded to the field for sample collection. Before start of the study, the parents, guardians and children were sensitized at their various quarters. A total of 500 children were randomly selected from the ten quarters in the study area by drawing from a list of homes with children less than 15 years of age. Consent forms were sent through the community health worker to parents and guardians of children in the selected homes seeking their consent/assent to participate in the study. Only the 454 children who presented a signed consent/assent form or verbal consent from parent or guardian took part in the study. The sample size for the study was calculated using the 85.4 % prevalence of malaria in children in the study area in 2006 [23]. The sample size was determined using the formula *n* = Z^2^pq/d^2^ [24] where *n* = the sample size required, z = 1.96: confidence level test statistic at the desired level of significance, p = 85.4 %: proportion of malaria prevalence, q = 1-p: proportion of malaria negative children and d = acceptable error willing to be committed. The minimum sample size was estimated as *n* = 192. This was adjusted to an optimum of at least 450 samples taking into consideration the recently reported decline in malaria parasite prevalence [1]. Before start of the study, the parents, guardians and children were educated at their various quarters. The investigation methods included clinical evaluation and laboratory investigations.

### Clinical evaluation

Clinical evaluation was carried out by trained medical personnel. The clinical evaluation consisted of: measurement of body temperature, examination of the spleen, measurement of blood pressure and heart rate. Axillary body temperature was measured using a digital thermometer. A child with a body temperature ≥37.5 °C was considered febrile. The tip of the spleen was felt by pressing the abdomen under the left coastal border and splenomegaly was graded according to the classification of Hackett on a scale of 1–5 [25, 26]. Children with a Hackett score of zero (0) were classified as normal; while those with a Hackett score of 1–2 and > 2 were classified as having mild splenomegaly and very large spleen respectively. An automatic arm sphygmomanometer (Pic indolor Diagnostic CS410) was used for non-invasive recording of blood pressure and heart rate. Systolic and diastolic blood pressure (mmHg) and heart rate (beats per min) were assessed according to the method of Bickley [27].

### Sample collection

Four (4) mL of venous blood was collected from each participant into sterile disposable syringes. A small portion was used for the preparation of thick and thin blood films. The remainder was dispensed into labelled ethelenediaminetetraacetate (EDTA) tubes, and transported in a cool box (temp 8–10 °C) to the Malaria Research Laboratory, University of Buea for further analysis.

### Laboratory methods

The thick and thin blood films prepared on glass slide at the time of blood sampling were stained with 10 % Giemsa and examined following standard protocols [28]. Parasite density was determined on the basis of number of parasites per 200 leukocytes on thick blood film with reference to participants white blood cell counts (WBC). If gametocytes were seen, the count was extended to 500 leukocytes [29]. Slides were considered positive when asexual forms and/or gametocytes of any *Plasmodium* species were observed on the blood film. Parasitaemia was classified as low (<1000 parasites/μL of blood), moderate (1000–4999 parasites/μL of blood) and high (≥5000 parasites/μL of blood). Slides were read by two independent parasitologists and in the case of any disparity they were read again by a third parasitologist. Blood samples in the EDTA tubes were transported to the St. Albert the Great Reference Medical Diagnostic Centre, for blood count analysis. The complete blood count analysis was obtained using an auto haematology analyser (TC Hemaxa 1000) following the manufacturer’s instructions. Parameters such as Hb concentration, Hct, RBC, WBC, granulocyte, lymphocyte and platelet counts, MCV, MCHC and MCH were obtained. Anaemia was defined as Hb <11 g/dL and was further classified into severe (Hb ≤ 6 g/dL), moderate (Hb = 6.1–8 g/dL) and mild (8.1–10.9 g/dL) anaemia [28]. Leucopenia was defined as WBC <4.5 × 10^9^/L. Thrombocytopaenia was defined as platelet count <150,000/μL.

### Statistical analysis

Data was entered into spread sheets using Microsoft Excel, validated and analysed with the Statistical Package for Social Sciences (SPSS) version 19 (IBM - SPSS, Inc, Chicago, IL, USA). Data were summarized into means and standard deviations (SD), and percentages were used in the evaluation of the descriptive statistics. Proportions were compared using the Chi-square test (*χ*^2^). The non parametric test, Kruskal-Wallis test, was used to compare the mean cardiovascular indices in the different age groups (≤5, 6–10 and 11–14 years) and malaria parasitaemia status (AP, ASP Pos, and ASP + Gam Pos). The mean temperature of children with different parasitaemic status was compared using analysis of variance (ANOVA). Gametocyte counts were log transformed before analysis. Pearson’s correlation coefficient was used to examine the relation between temperature and heart rate. Cardiovascular indices with a *P*-value ≤0.2 in the univariate analyses were entered into a multiple linear regression (MLR) model with each haematological variable, systolic and diastolic blood pressure as the dependent variable. The MLR (enter) models were run after controlling for age to examine the influence of the following independent variables; sex, temperature, asexual parasitaemia, gametocyte carriage and spleen size. All 454 participants were included in the model. Significant levels were measured at 95 % confidence interval (CI) with significant differences set at *P* < 0.05.

### Ethical considerations

Administrative clearance was obtained from the South West Regional Delegation of Public Health. Further authorization was obtained from the chief and quarter heads of the Muea community. The institutional review board hosted by the Faculty of Health Sciences, University of Buea issued the ethical clearance document for the study after reviewing the study protocol, participant’s information sheet and assent forms. During sensitisation at the beginning of the study, the protocol was explained and the benefits of participating in the study highlighted. Assent forms were then given to parent/guardian requesting the approval of their children’s participation in the study. For parents/guardians who were unable to read or write, the information was read and explained to them and verbal consent requested. The verbal consent was documented by the team on the assent form. The participation of the children was voluntary and only those with signed assent forms were enrolled into the study.

## Results

### Baseline characteristics of the study population

A total of 454 children with a mean age of 6.7 ± 3.4 years of both sexes residing in Muea in the Mount Cameroon area were evaluated for the influence of gametocyte carriage on splenic status and cardiovascular indices. The overall prevalence of malaria parasites in the study population was 36.6 % (CI = 32.2–41.1 %). Gametocytaemia, splenomegaly, fever, leucopaenia and thrombocytopaenia were detected in 7.3, 14.3, 21.6, 24.6 and 18 % of the children respectively, as shown in Table 1.

**Table 1:** Characteristics of study population

Characteristic		All
Mean age in years (Range)		6.7 ± 3.4 (0.5–14)
Age groups (years)	≤5 (%)	177 (39)
	6–10 (%)	205 (45.2)
	>10 (%)	72 (15.8)
Sex	Male (%)	211 (46.5)
	Female (%)	243 (53.5)
Prevalence (%) of gametocytaemia		7.3
Prevalence (%) of splenomegaly		14.3
Average enlarged spleen (AES)		2.8 ± 1.5
Mean WBC count × 10^9^/L (Range)		8.01 ± 3.2 (1.6–33.1)
Prevalence (%) of fever		21.6
Prevalence (%) of leucopaenia		24.6
Mean lymphocyte count × 10^9^/L (Range)		4.38 ± 2.02 (0.8–12.4)
Mean RBC count × 10^12/^ L (Range)		4.55 ± 0.69 (1.1–8.6)
Mean haemoglobin in g/dL (Range)		11.6 ± 1.9 (2.4–21.1)
Mean platelet count × 10^9^/ L (Range)		219.91 ± 87.72 (27–573)
Prevalence (%) of thrombocytopenia		18
Mean systolic blood pressure in mmHg (Range)		101.56 ± 16.7 (72–182)
Mean diastolic blood pressure in mmHg (Range)		66.61 ± 15.9 (41–156)
Mean heart rate in beats/min (Range)		100.21 ± 17.7 (54–160)

Low, moderate and high malaria parasitaemia were common in 56.5 % (CI = 48.8–63.9 %), 29.8 % (CI = 23.3–37.3 %) and 13.7 % (CI = 9.2–19.8 %) of the children respectively. The prevalence of low, moderate and high malaria parasitaemia were comparable (*P* = 0.55) in children who were ASP Pos (56.6, 30.9, 12.2 %) and those with ASP + Gam (56.0, 24.0 and 20.0 % respectively). Although not statistically significant (*P* = 0.26), children with ASP only had a higher geometric mean parasite density/μL of blood (859) when compared with those with ASP + Gam (639).

### Gametocytaemia and age effect

Out of the 454 children examined, 33 (7.3 %, CI = 5.2–10.0 %) were gametocyte positive with a geometric mean gametocyte density (GMGD) of 56/μL of blood (range = 15–306 gametocytes/μL). The GMGD/μL was comparable (*P* = 0.56) in males (51) and females (61). Although children of the ≤ 5 years age group had the highest GMGD/μL of blood (69) when compared with the 10–14 years (30) and the 6–10 years (51) age groups, the differences were however not statistically significant (*P* = 0.12).

As revealed in Table 2, the proportion of children in the community who were AP, ASP Pos and ASP + Gam Pos did not vary significantly in the various age groups. The systolic blood pressure in children of the 6–10 years age group was significantly lowest in those with ASP + Gam (87.1 ± 6.1 mmHg) than their AP (104.2 ± 18.5 mmHg) and ASP Pos (99.6 ± 16.2 mmHg) counterparts. Malaria parasitaemic status did not have a significant effect on the diastolic blood pressure and heart rate in the different age groups.

**Table 2:** Effect of age and malaria parasitaemia status on some cardiovascular indices

Parameter	Category	Malaria parasitaemia status			*P*- value
		AP	ASP Pos	ASP + Gam Pos	
N		285	136	33	
Age group in Years % (n)	≤ 5	38.6 (110)	37.5 (51)	45.5 (15)	0.51
	6–10	46.7 (133)	42.6 (58)	45.5 (15)	
	11–14	14.7 (42)	19.9 (27)	9.1 (3)	
Mean (SD) Systolic blood pressure (mmHg)	≤ 5	97.0 (11.7)	103.9 (25.6)	104.1 (18.6)	0.57
	6–10	104.2 (18.5)	99.6 (16.2)	87.1 (6.1)	<0.001
	11–14	101.0 (12.3)	104.6 (11.8)	98.0 (13.9)	0.33
Mean (SD) Diastolic blood pressure (mmHg)	≤ 5	63.5 (9.8)	72.2 (27.7)	68.0 (23.4)	0.79
	6–10	67.7 (17.9)	66.4 (13.6)	57.2 (9.1)	0.09
	11–14	64.5 (10.2)	69.4 (10.7)	65.7 (9.3)	0.17
Mean (SD) Heart rate (Beats/min)	≤ 5	107.6 (19.4)	112.5 (24.9)	101.6 (22.8)	0.29
	6–10	99.3 (15.8)	101.2 (13.4)	112.6 (21.3)	0.21
	11–14	89.0 (11.9)	91.8 (13.6)	95.3 (2.3)	0.37

### Fever, anaemia and splenomegaly

The mean temperature in the study population was 37.0 ± 0.64 ^o^C with a range of 35.8–40.4 ^o^C. A significant positive correlation (*r* = 0.27, *P* = 0.028) was observed between temperature and heart rate. The prevalence of fever was comparable (*P* = 0.70) in children of the age group ≤ 5 (20.5 %, 36), 6–10 (23.4 %, 48) and > 10 years (19.4 %, 4) irrespective of the malaria parasitaemia status. However, the prevalence of fever was significantly highest (*P* = 0.03) in children who were ASP + Gam Pos (39.4 %) than their equivalents as revealed in Table 3.

**Table 3:** Effect of malaria parasitaemia status on temperature/fever and anaemia

Parameter	Malaria parasitaemia status			Test
	AP (n)	ASP Pos (n)	ASP + Gam Pos (n)	*P*- value
N	285	136	33	
Mean Temperature	37.0 ± 0.6	37.0 ± 0.7	37.5 ± 0.8	9.66^a^ <0.001
Prevalence (%) of Fever	19.0 (54)	22.8 (31)	39.4 (13)	7.08^b^ 0.025
Prevalence (%) of Anaemia	21.4 (61)	38.2 (52)	54.5 (18)	24.15^b^ <0.001

^a^F test

^b^-*χ* ^2^ test

The prevalence of anaemia in the study population was 28.9 % (131), while mild, moderate and severe anaemia was prevalent in 24.2 % (110), 2.6 % (12) and 2.0 % (9) of the children respectively. Anaemia prevalence was significantly highest (*P* < 0.001) in children with ASP + Gam (54.5 %) when compared with those AP (21.4 %) and ASP Pos (38.2 %).

**Fig. 1:**
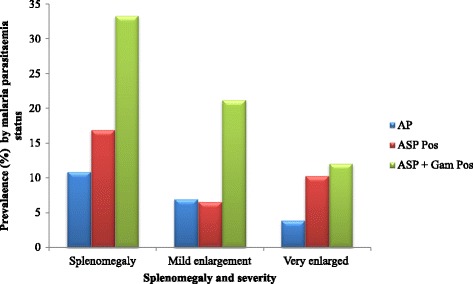
Prevalence and severity of splenomegaly as affected by malaria parasitaemia status

Out of the 454 children examined, 65 (14.3 %) had enlarged spleens with an AES of 2.8 ± 1.5. The AES was comparable (*P* = 0.68) in the different age groups (≤5 years = 2.6 ± 1.3; 6–10 years = 2.9 ± 1.5; 11–14 years = 3.0 ± 1.7). Similarly, AES was comparable (P = 0.21) in AP (2.6 ± 1.4), ASP Pos (3.3 ± 1.5) and ASP + Gam Pos (2.6 ± 1.4). Overall, the prevalence of mild enlargement was 7.9 % (36), while 6.4 % (29) of the children had very enlarged spleens. Mild enlargement and very enlarged spleen were significantly highest (*P* = 0.001) in children with ASP + Gam (21.2 and 12.1 % respectively) than their counterparts. Likewise children with ASP + Gam had significantly highest (*P* = 0.001) prevalence of splenomegaly (33.3 %, 11) as shown in Fig. 1.

**Fig. 2:**
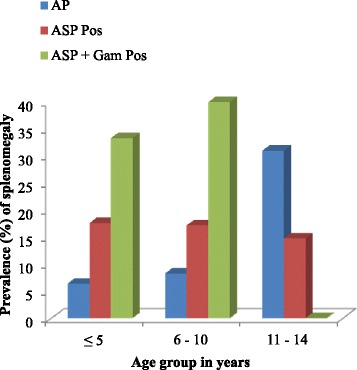
Prevalence of splenomegaly in the different age groups as affected by malaria parasitaemia status

Children with ASP + Gam in the < 5 years and the 6–10 years age groups had significantly highest (*χ*^2^ = 11.37 *P* = 0.003; *χ*^2^ = 13.13 *P* = 0.001) prevalence of splenomegaly (33.3 and 40 % respectively) in comparison with the aparasitaemic and those with asexual parasites only as shown in Fig. 2.

**Fig. 3:**
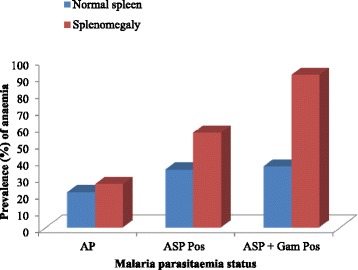
Prevalence of anaemia as affected by splenic and malaria parasitaemia status

A comparison of prevalence of anaemia revealed a higher prevalence of anaemia in children with splenomegaly than in those with normal spleen (Fig. 3). However, only the difference in prevalence of anaemia in those ASP + Gam Pos (Normal spleen = 36.4 % and splenomegaly = 90.9 %) was statistically significant (*P* = 0.003).

### Influence of gametocyte carriage on cardiovascular indices

As shown in Table 4, children who were ASP + Gam Pos had significantly lower Hct (*P* < 0.001), Hb levels (*P* < 0.001), RBC counts (*P* = 0.029), platelet counts (*P* = 0.02) and systolic blood pressure (*P* = 0.049) when compared with their AP and ASP Pos counterparts. Although not statistically significant, the mean WBC and lymphocyte counts and diastolic blood pressure were lower in children who were ASP + Gam Pos than those AP and those ASP Pos only. The mean MCV, MCH, MCHC and MPV were comparable in all three groups of children.

**Table 4:** Comparison of mean cardiovascular indices in AP, ASP Pos and ASP + Gam Pos children

Parameter	Malaria parasitaemia status			*P*-value
	AP	ASP Pos	ASP + Gam Pos	
	Mean (SD)	Mean (SD)	Mean (SD)	
N	285	136	33	
WBC x 10^9^/L	8.1 (3.2)	8.0 (3.2)	7.3 (3.8)	0.09
Lymphocyte x 10^9 ^/L	4.4 (2.0)	4.4 (2.1)	3.7 (1.8)	0.16
Granulocytes x 10^9^/L	2.4 (1.5)	2.5 (1.8)	2.1 (1.2)	0.80
MCV (fl)	79.0 (6.2)	78.4 (6.8)	78.2 (5.6)	0.22
MCH (Pg)	25.9 (2.7)	25.7 (6.8)	25.3 (1.9)	0.13
MCHC (g/L)	32.7 (2.2)	32.8 (1.5)	32.5 (1.7)	0.48
Hct (%)	35.8 (5.1)	34.7 (5.0)	31.7 (6.1)	<0.001
Hb (g/dL)	11.8 (1.9)	11.4 (1.8)	10.4 (2.2)	<0.001
RBC x 10^9^/L	4.6 (0.6)	4.6 (0.8)	4.2 (0.9)	0.029
Platelet x10^9^/L	229.8 (87.5)	206.3 (83.3)	200.7 (79.3)	0.016
MPV (fL)	9.2 (3.1)	9.0 (0.8)	8.8 (0.9)	0.33
Heart rate (Beats/min)	99.5 (17.2)	100.7 (18.1)	105.7 (20.7)	0.48
Systolic blood pressure (mmHg)	102.0 (16.3)	102.0 (17.9)	95.1 (14.8)	0.049
Diastolic blood pressure (mmHg)	66.2 (15.1)	68.5 (17.3)	62.5	0.097

### Influence of spleen size, sex, gametocyte and trophozoite counts on haematological parameters

After controlling for the effect of age on the independent variables, sex had a significant influence on MCV (*P* = 0.03). The size of the spleen significantly influenced the Hb (*P* = 0.01), Hct (*P* = 0.01) and RBC (*P* = 0.001). Temperature had a significant influence on Hb (*P* = 0.01), Hct (*P* = 0.03), lymphocyte counts (*P* = 0.02), MCH (*P* = 0.01) and heart beats/min (*P* < 0.001). The presence of asexual parasitaemia significantly influenced the MCV (*P* = 0.03), MCH (*P* = 0.03) and heart beats/min (0.03) while gametocytaemia significantly influenced the Hb (*P* < 0.001), Hct (*P* < 0.001), RBC (*P* < 0.001) and systolic blood pressure (*P* < 0.05) as shown in Table 5.

**Table 5:** Multiple linear regression analyses examining the influence of each independent variable on the dependent variable

Dependent variable	Independent Variable	β value	*P* value	R^2^
Hb g/dL	Spleen size	−0.12	0.01	0.1
	Temperature	−0.13	0.01	
	Asexual parasitaemia	−0.08	0.08	
	Gametocytaemia	−0.17	<0.001	
	Sex	0.001	0.98	
Hct %	Spleen size	−0.13	0.01	0.1
	Temperature	−0.10	0.03	
	Asexual parasitaemia	−0.08	0.10	
	Gametocytaemia	−0.17	<0.001	
	Sex	−0.03	0.58	
RBC × 10^12^/L	Spleen size	−0.14	0.01	0.1
	Temperature	−0.04	0.46	
	Asexual parasitaemia	−0.02	0.69	
	Gametocytaemia	−0.16	0.001	
	Sex	0.042	0.39	
Lymphocytes	Spleen size	−0.05	0.36	0.03
	Temperature	−0.12	0.02	
	Asexual parasitaemia	−0.06	0.76	
	Gametocytaemia	0.02	0.21	
	Sex	−0.06	0.26	
MCV	Spleen size	−0.01	0.91	0.03
	Temperature	−0.01	0.21	
	Asexual parasitaemia	−0.11	0.03	
	Gametocytaemia	0.01	0.86	
	Sex	−0.11	0.03	
MCH	Spleen size	−0.14	0.78	0.03
	Temperature	−0.13	0.01	
	Asexual parasitaemia	−0.12	0.03	
	Gametocytaemia	−0.02	0.69	
	Sex	−0.04	0.47	
Systolic blood pressure	Spleen size	−0.02	0.67	0.03
	Temperature	0.08	0.12	
	Asexual parasitaemia	−0.04	0.45	
	Gametocytaemia	−0.12	0.03	
	Sex	−0.04	0.53	
Heart beats/min	Spleen size	0.08	0.13	0.01
	Temperature	0.24	<0.001	
	Asexual parasitaemia	0.11	0.03	
	Gametocytaemia	0.08	0.14	
	Sex	−0.08	0.13	

## Discussion

Infection with *P. falciparum* is very likely to result in symptoms that include fever and splenomegaly. Even though splenomegaly is one of the main clinical markers of endemicity of malaria in *P. falciparum* transmission areas [19], it is frequently absent during acute infection in non-immune subjects [7, 30]. The proportion of fever and splenomegaly in communities in malaria endemic regions requires investigation as the proportion of individuals with these conditions who report to health facilities harbouring infection may be higher. This paper in addition investigates the influence of gametocyte carriage on blood pressure, heart rate and haematological indices in children with malaria parasitaemia, living in a *meso* endemic community in the Mount Cameroon area.

The overall prevalence of fever (21.6 %) is significantly lower than that reported by Sumbele *et al.* [23] in the same community (28.2 %) in 2006 and comparable to the report of Songue *et al.* [31] during the rainy season in children in communities along the Chad Cameroon pipeline. The decrease in prevalence has been attributed to the intensification of malaria control measures. However, consistent with other findings [32], the prevalence of fever was higher in children with malaria parasitaemia. The relationship between fever and *P. falciparum* parasitaemia depends strongly on season which may thus affect the malaria-attributable fraction of fever cases [33]. Never the less, fever per se occurred less often in children who were aparasitaemic and malaria parasitaemic children who in addition carried gametocytes recorded the highest prevalence of fever.

Parasite prevalence and level of exposure are closely related and clinically immune infections have been reported to have lower gametocytemia as a result of having anti-parasite immunity, conferring protection against high-density parasitaemia [4]. The lack of anti-parasite immunity is evident in children of the ≤ 5 years age group who had the highest geometric mean gametocytes/μL of blood than their counterparts. The overall prevalence of gametocytes in the study population was lower (7.3 %) than the 17.49 % reported by Kimbi *et al*. [34] in children in the same area. On the other hand, the gametocyte density was higher (56/μL of blood) than that (23/μL of blood) reported by Kimbi *et al.* [34]. The higher gametocyte density is unusual in an era where amodiaquine-atesunate, an artemisin combination therapy which is gametocidal and significantly lowers gametocyte prevalence and density following treatment [35] is used as the first line treatment of non severe malaria in Cameroon. This probably brings to light the persistence of low grade malaria parasitaemia in some of the children resulting in the development of gametocytes. The fact that a number of the children with asexual parasites concurrently harboured gametocytes has direct implications for transmissibility. These children probably did not seek treatment and were thus able to carry malaria parasites for longer duration resulting in the development of gametocytes and the ensuing consequences.

The lowered WBC and lymphocyte counts noticed in children harbouring gametocytes and asexual parasite is unusual as the aparasitaemic and those harbouring asexual parasite only had similar mean WBC and lymphocyte counts. However, the observed malaria associated decrease in WBC and lymphocyte counts is in line with the decrease in lymphocyte count in untreated malaria reported by Sumbele *et al*. [16]. Furthermore, the temperature of the child significantly influenced the lymphocyte counts as revealed in the multivariate analysis. At the first look, this is unexpected as lymphocytes, particularly T cells, play a major role in immunity against *P. falciparum* malaria by releasing pro- and anti-inflammatory cytokines such as TNF-α, interferon-γ and other cytokines, and activating other inflammatory cells. However, excessive secretion of the pro-inflammatory cytokines contributes to disease severity [12, 36].

Findings from the multiple linear regression analysis revealed that the presence of gametocytes significantly influenced the Hb, Hct, RBC and systolic pressure. In like manner, a comparison of haematological variables revealed these variables to be significantly lowest in children harbouring both gametocytes and asexual parasites. The reduction in these red cell indices in gametocyte positive children is not unusual as Sumbele *et al*. [8] and Nacher *et al*. [37] reported a strong link between anaemia and gametocyte carriage. The destruction of the oxygen transporter (Hb) and tissue hypoxia leading invariably to erythrocyte destruction is conducive for gametogenesis [3].

One of the additive significant influences of gametocyte carriage is the lowered systolic blood pressure observed in children who had both asexual parasites and gametocytes when compared with those aparasitaemic and those harbouring only asexual parasites. This may be associated to the fever observed in this group of children. Fever and excessive sweating, accompanied with lack of rehydration, lead to continuous dehydration. During hyperthermia, one of the mechanisms involved in regulating the temperature by the body is vasodilation. This is caused by inhibition of the sympathetic centres in the hypothalamus [38]. More also, the host immune reaction against malaria parasites involves pro- and anti-inflammatory cytokines as well as mediators like nitric oxide [12], a major down-regulator of vascular tone [39–41]. The loss of water and the decrease of peripheral resistance (vasodilation) lead to a reduction of blood pressure. Furthermore, *P. falciparum* infection is known to cause low platelet levels and disseminated intravascular coagulation with bleeding [13], resulting in low blood pressure. The significantly low mean platelet count observed in children harbouring gametocytes in comparison with the aparasitaemic and those with asexual parasitaemia only in the univariate analysis was of no consequence in the multivariate analysis.

Observation from the study revealed the presence of mild and very enlarged spleens in the study population. Splenic enlargement has been highlighted as a consequence of malaria parasitaemia [42] and multiple parasitic aetiologies [5]. A palpable spleen may arise due to congestion of RBCs, increase in the relative number of macrophages in the spleen, hence leading to an increase in spleen weight associated with expansion of both the red and white pulp [43]. Splenomegaly was significantly highest in children harbouring both asexual parasite and gametocytes than their counterparts, especially those in the ≤5 years and 6–10 years age groups. In fact, mild and very enlarged spleens were significantly highest in children with ASP + Gam than their AP and ASP Pos counterparts. Further observation from the study revealed the significantly highest prevalence of anaemia to occur in children with splenomegaly concurrently harbouring both asexual parasites and gametocytes. In Cameroon, anaemia remains a major public health problem [8, 32] and malaria has been frequently associated with haemolysis, anaemia, decreased erythropoiesis and splenomegaly [8, 44]. The contribution of splenic enlargement to early anaemia of acute malaria through sequestering RBCs in the spleen have been highlighted [38, 45]. Additionally, findings from another study by Crookston *et al*. [46] reported children who experience anaemia and have splenomegaly are more likely to present asymptomatic malaria. This probably accounts for the prevalence of splenomegaly and anaemia observed in the community where a large proportion of the children with malaria parasitaemia were asymptomatic.

Findings from the study demonstrated a significant influence of the spleen size on red cell indices such as the Hb level, Hct, and RBC while, temperature in addition to the Hb and Hct significantly influenced the MCH. The effects may be attributed to the role of the spleen in clearing infected [38, 47] and non-infected red blood cells from the circulation [38] while fever and the inflammatory reaction may shorten RBC survival [48]. In line with previous studies [3, 4], a palpable spleen was revealed to be an independent risk factor of anaemia and anaemia is more pronounced in patients with malaria than in other systemic infections [48]. As anaemia progressively worsens, platelet counts decrease with accompanying splenomegaly suggesting hypersplenism may, at least in part, contribute to thrombocytopenia [49]. This is in line with the findings of this study as children with splenomegaly were 1.1 (CI = 0.93–1.30) times at odds of having thrombocytopaenia than those with normal spleen.

Although not significant, the heart rate was higher in gametocyte positive children than those aparasitaemic and those having asexual parasites only. As well, the temperature of the child was significantly associated with the heart rate. The significant association of temperature with the heart rate is not unexpected as an increase of only 1 °C raises the heart rate by about 10 beats per minute. This explains the faster heart beats observed in cases with fever [50]. The higher heart rate in children infected with both asexual and sexual stages of malaria parasites may be a feedback of the heart to the decrease of the cardiac output (CO). In effect, in patients with malaria, the pre-load may decrease or the after-load increases and this might contribute to a decreased CO [11, 12]. Also, sequestered parasitized RBCs obstruct the microvasculature causing tissue hypoxia [51], secondary to cytoadhesion of parasite infected red blood cells (RBCs) to vascular endothelium [11, 52]. In addition, contact of platelet membrane damages endothelial lining of vessels [53] and the sequestered RBCs undergo haemolysis, increasing anaemia and reduced tissue oxygen provision. Resting heart rate is higher in people with anaemia due to the decreased number of RBCs present in circulation [54]. Tachycardia is one of the most common symptoms of iron-deficiency anaemia. These results are in line with other findings [13, 14] showing that impairment of cardiovascular parameters is common in *P. falciparum* malaria as the disease gets toward complication. The observed increase of the heart rate could help balance the provision of oxygen to the tissues.

In addition to the gametocytaemia, increased spleen size, the trophozoite count also had significant influence on haematological indices such as MCV and MCH and cardiovascular indices like the heart beats/min. It is worth noting that despite all variations in the hemodynamic parameters, majority of the values remained within normal ranges. However, our findings can serve as a relevant warning signal, highlighting the hidden danger of the impact of gametocyte carriage, asymptomatic malaria parasitaemia and uncomplicated malaria on the cardiovascular system. The fact that some children did not even display signs of malaria infection is a strong proof that regular investigations are invaluable in malaria endemic areas. The more infections are persistent in an area of transmission, the more risky the population will be, irrespective of whether the patient has asymptomatic or uncomplicated malaria.

## Conclusions

The results showed that in a malaria endemic setting such as the Mount Cameroon area, gametocyte carriage significantly influenced the prevalence of fever, splenomegaly and some cardiovascular indices. In effect, children concurrently having asexual parasitaemia and gametocytes had significantly lower, Hct, Hb levels, RBC and platelet counts and systolic blood pressure when compared with gametocyte negative children. Further investigations with ultrasound cardiography will surely bring out more information on the cardiac profile of these children.

## Abbreviations

Ap: Aparasitaemic
ASP: Asexual parasitaemia
CO: Cardiac output
CI: 95 % confidence interval
Gam: Gametocytes
GMGD: Geometric mean gametocyte density
Hb: Haemoglobin
Hct: Haematocrit
MCH: Mean corpuscular haemoglobin
MCHC: Mean corpuscular haemoglobin concentration
MCV: Mean corpuscular volume
MLR: Multiple linear regression
RBC: Red blood cell
SD: Standard deviation
WBC: White blood cell

## References

[1] World Health Organisation. World malaria report 2014, Geneva. World Health Organisation. http://www.who.int/malaria/publications/world_malaria_report_2014/report/en/9789241564830_eng.pdf.

[2] Okiro EA, Snow RW (2010). Relationship between reported fever and *Plasmodium falciparum* infection in African children. Malar J.

[3] Bousema T, Drakeley C (2011). Epidemiology and infectivity of *Plasmodium falciparum* and *Plasmodium vivax* gametocytes in relation to malaria control and elimination. Clin Microbiol Rev.

[4] Doolan DL, Dobano C, Baird JK (2009). Acquired immunity to malaria. Clin Microbiol Rev.

[5] Mboera LEG, Senkoro KP, Rumisha SF, Mayala BK, Shayoa EH, Mloz MRS (2011). *Plasmodium falciparum* and helminth co-infections among school children in relation to agro-ecosystems in Mvomero District, Tanzania. Acta Trop.

[6] Buffet PA, Safeukui I, Deplaine G, Brousse V, Prendki V, Thellier M (2011). The pathogenesis of *Plasmodium falciparum* malaria in humans: insights from splenic physiology. Blood.

[7] Price RN, Simpson JA, Nosten F, Luxemburger C, Hkirjaroen L (2001). Factors contributing to anaemia after uncomplicated *falciparum malaria*. Am J Trop Med Hyg.

[8] Sumbele IUN, Samje M, Nkuo-Akenji T (2013). A longitudinal study on anaemia in children with *Plasmodium falciparum* infection in the Mount Cameroon region: prevalence, risk factors and perceptions by caregivers. BMC Infect Dis.

[9] Grenfell P, Fanello CI, Magris M, Goncalves J, Metzger WG (2008). Anaemia and malaria in Yanomami communities with differing access to healthcare. Trans R Soc Trop Med Hyg.

[10] Korenromp EL, Amstrong-Schillinberg JRM, Williams BG, Nahlen BL, Snow RW (2004). Impact of malaria control in childhood anaemia in Africa-a quantitative review. J Trop Med Int Health.

[11] Mishra KS, Behera KP, Satpathi S (2013). Cardiac involvement in malaria. An overlooked important complication. J Vector Borne Dis.

[12] Herr J, Mehrfar P, Schmiedel S, Wichmann D, Brattig NW, Burchard GD (2011). Reduced cardiac output in imported *Plasmodium falciparum* malaria. Malar J.

[13] Anigbogu CN, Adigun SA (1996). Blood pressure, heart rate and autonomic reflexes in *P. bergei* malaria infection. Nig Q J Hosp Med.

[14] Anigbogu CN, Olubowale OA (2002). Effects of malaria on BP, HR, electrocardiogram and cardiovascular response to change in posture. Nig Q J Hosp Med.

[15] Facer CA (1994). Hematological aspects of malaria. Infection and Haematology.

[16] Sumbele IUN, Nkuo-Akenji T, Samje M, Ndzeize T, Ngwa EM, Titanji VPK (2010). Haematological changes and recovery associated with treated and untreated *Plasmodium falciparum* infection in children in the Mount Cameroon Region. J Clin Med Res.

[17] Kimbi HK, Nformi D, Ndamukong KJ (2005). Prevalence of malaria among school children in an urban and rural area in the mount Cameroon region. Cent Afr J Med.

[18] Nkuo-Akenji TK, Sumbele I, Mankah EN, Njunda AL, Samje M, Kamga L (2008). The burden of malaria and malnutrition among children less than 14 years of age in a rural village of Cameroon. AJFAND.

[19] Snow RW, Omumbo JA, Lowe B (1997). Relationship between severe malaria morbidity in children and level of *Plasmodium falciparum* transmission in Africa. Lancet.

[20] Janka JJ, Koit OA, Traoré B, Traoré JM, Mzayek F, Sachdev V (2010). Increased pulmonary pressures and myocardial wall stress in children with severe malaria. J Infect Dis.

[21] Yacoub S, Lang HJ, Shebbe M, Timbwa M, Ohuma E, Tulloh R (2010). Cardiac function and hemodynamics in Kenyan children with severe malaria. Crit Care Med.

[22] Nkuo-Akenji T, Ntonifor NN, Kimbi HK, Abongwa EL, Ching JK, Ndukum MB (2005). The epidemiology of malaria in Bolifamba, a rural community in the eastern slopes of mount Cameroon: seasonal variation in the parasitological indices of transmission. Ann Trop Med Parasitol.

[23] Sumbele IUN, Ning TR, Bopda OSM, Nkuo-Akenji T (2014). Variation in malariometric and red cell indices in children in the Mount Cameroon area following enhanced malaria control measures: evidence from a repeated cross-sectional study. Malar J.

[24] Bryan FJ (1992). The design and analysis of research studies, University of Otago.

[25] Gilles HM (1997). Pathology of malaria: Handbook of malaria infection in the tropics.

[26] Ogilvie C, Evans CC. Splenomegaly. In Wratt G: symptoms and signs in tropical disease. Chamberlaine’s symptoms and signs in clinical medicine. 12th ed. Oxford: Butterwoth-Heineman; 1997 pp. 315-324.

[27] Bickley LS (2002). Bates’ guide to physical examination and history taking.

[28] Cheesbrough M (2009). District laboratory practice in Tropical Countries. Part 1. Cambridge low price editions.

[29] Trape JF (1985). Rapid evaluation of malaria parasite density and standardization of thick smear examination for epidemiological investigations. Trans R Soc Trop Med Hyg.

[30] Giha HA, Elghazali G, A-Elgadir TM, A-Elbasit IE, Elbashir MI (2009). Severe malaria in an unstable setting: clinical and laboratory correlates of cerebral malaria and severe malarial anemia and a paradigm for a simplified severity scoring. Eur J Clin Microbiol Infect Dis.

[31] Songue E, Tagne C, Mbouyap P, Essomba P, Moyou–Somo R (2013). Epidemiology of Malaria in three Geo-Ecological Zones along the Chad-Cameroon Pipeline. Am J Epidem Infect Dis.

[32] Achidi EA, Apinjoh TO, Anchang-Kimbi JK, Mugri RN, Ngwai AN, Yafi CN (2012). Severe and uncomplicated *falciparum* malaria in children from three regions and three ethnic groups in Cameroon: prospective study. Malar J.

[33] Dicko A, Mante C, Kouriba B, Sagara I, Thera MA, Doumbia S (2005). Season, fever prevalence and pyrogenic threshold for malaria disease definition in an endemic area of Mali. Trop Med Int Hlth.

[34] Kimbi HK, Keka FC, Nyabeyeu HN, Ajeagah HU, Tonga CF, Lum E (2012). An update of asymptomatic falciparum malaria in school children in Muea, Southwest Cameroon. J Bacteriol Parasitol.

[35] Stepniewska K, Price RN, Sutherland CJ, Drakeley CJ, von Seidlein L, Nosten F (2008). *Plasmodium falciparum* gametocyte dynamics in areas of different malaria endemicity. Malar J.

[36] Biemba G, Gordeuk VR, Thuma P, Weiss G (2000). Markers of inflammation in children with severe malarial anaemia. J Trop Med Int Health.

[37] Nacher M, Singhasivanon P, Silachamroon U, Treeprasertsuk S, Tosukhowong T, Vannaphan S (2002). Decreased haemoglobin concentration, hyperparasitaemia and severe malaria as associated with increase *Plasmodium falciparum* gametocyte carriage. J Parasitol.

[38] Guyton AC, Hall JE. A textbook of medical Physiology. 11th ed. Philadelphia, Pennsylvania 19103–2899: Elsevier Saunders; 2006.

[39] Calver A, Collier J, Vallance P (1993). Nitric oxide and the control of human vascular tone in health and disease. Eur J Med.

[40] Bopda MOS, Longo F, Bella NT, Edzah OPM, Taïwe SG, Bilanda DC (2014). Antihypertensive activities of the aqueous extract of *Kalanchoe pinnata* (Crassulaceae) in high salt-loaded rats. J Ethnopharmacol.

[41] Qu Z, Zhang J, Gao W, Chen H, Guo H, Wang T (2014). Vasorelaxant effects of Cerebralcare Granules are mediated by NO/cGMP pathway, potassium channel opening and calcium channel blockade in isolated rat thoracic aorta. J Ethnopharmacol.

[42] Bryceson A, Fakunle YM, Fleming AF, Crane G, Hutt MS, de Cock KM (1983). Malaria and splenomegaly. Trans R Soc Trop Med Hyg.

[43] Urban BC, Ferguson DJ, Pain A (1999). *Plasmodium falciparum*-infected erythrocytes modulate the maturation of dendritic cells. Nature.

[44] Adedapo AD, Falade CO, Kotila RT, Ademowo GO (2007). Age as a risk factor for thrombocytopaenia and anaemia in children treated for acute uncomplicated malaria. J Vector Borne Dis.

[45] Menendez C, Fleming AF, Alonso PL (2000). Malaria and Anaemia. J Parasitol.

[46] Crookston BT, Alder SC, Boakye I, Merril RM, Amuasi JH, Porucznik CA (2010). Exploring the relationship between chronic undernutrition and asymptomatic malaria in Ghanaian children. Malar J.

[47] Jenkins MK, Khoruts A, Inqulli E, Mueller DL, McSorley SJ, Reinhardt RL (2001). In vivo activation of antigen-specific CD4 T cells. Annu Rev Immunol.

[48] Ekval H (2003). Malaria and anemia. Curr Opin Hematol.

[49] Novelli EM, Hittner JB, Davenport GC, Ouma C, Were T, Obaro S (2010). Clinical predictors of severe malarial anaemia in a holoendemic *Plasmodium falciparum* transmission area. Brit J Haematol.

[50] Campbell NA, Reece JB, Urry LA, Cain ML, Wasserman SA, Minorsky PV (2008). Biology.

[51] Dondorp AM, Ince C, Charunwatthana P (2008). Direct in vivo assessment of microcirculatory dysfunction in severe *falciparum* malaria. J Infect Dis.

[52] Kyes S, Horrocks P, Newbold C (2001). Antigenic variation at the red cell surface in malaria. Annu Rev Microbiol.

[53] Essien EM (1989). The circulating platelet in acute malaria infection. Br J Haematol.

[54] Katz SD, Maskin C, Jondeau G, Cocke T, Bekowitz R, LeJemtel T (2000). Near-maximal fractional oxygen extraction by active skeletal muscle patients with chronic heart failure. J Appl Physiol.

